# Examination of *S*-Locus Regulated Differential Expression in *Primula vulgaris* Floral Development

**DOI:** 10.3390/plants7020038

**Published:** 2018-05-02

**Authors:** Benjamin Burrows, Andrew McCubbin

**Affiliations:** School of Biological Sciences, Washington State University, Pullman, WA 99164, USA; bburrows@wsu.edu

**Keywords:** *Primula*, heteromorphic self-incompatibility, RNA-seq, *S*-locus, flower development

## Abstract

Recent findings on the molecular basis of heteromorphic self-incompatibility in *Primula* have shown that the controlling self-incompatibility (*S*)-locus is not allelic, but is instead a small hemizygous region of only a few genes in the thrum genotype. How these genes alter the development of floral morphology and the specificity of self-incompatibility is still not completely clear. In order to start to identify genes regulated by the *S*-locus and elucidate the large-scale biological processes affected, we used RNA-seq data from floral buds of heteromorphic *P. vulgaris* pin (long style, short anthers) and thrum (short style, long anthers) morphs at early and late developmental time points. Differential expression between the two morphs was assessed at both time points and Gene Ontology term analyses of these gene sets were conducted. Our findings suggest that the *S*-locus regulates a large number of genes outside its physical bounds and likely sets up a cascade of expression changes. Additionally, we found evidence to suggest that there may be a timing difference in pollen development between the morphs, with pin pollen development proceeding earlier than thrum pollen development. This finding provides insight into how morphological differences in pollen between the morphs may be established, but intriguingly, could also be related to the self-incompatibility phenotype.

## 1. Introduction

Heteromorphic self-incompatibility (HSI) is one of many breeding systems that have evolved in the angiosperms to prevent inbreeding and promote out-crossing [1]. *Primula vulgaris* possesses a classical HSI breeding system that combines distyly with physiological self-incompatibility and has historically been of considerable interest to geneticists and evolutionary biologists (reviewed in [2,3]). *P. vulgaris* possesses two hermaphrodite floral mating types. The long-styled morph, termed “pin”, has the receptive female stigma positioned near the corolla tube mouth and the anthers approximately halfway down the corolla tube. The other mating type, the short-styled “thrum”, has reciprocal positioning of these sexual organs, relative to the pin morph. Only inter-morph pollinations are compatible and result in fertilization [4,5]. These morphological differences promote cross-pollination between, and reduce pollination within, mating types, but do not fully prevent within-mating type fertilization—a task performed by the self-incompatibility system [6,7].

Classical genetic analyses of HSI in *Primula* identified a single diallelic genetic locus (the *S*-locus) controlling the phenomenon. The short-styled thrum morph was considered heterozygous (*Ss*) for the *S*-locus, while the long-styled pin morph was considered homozygous recessive (*ss*) [8]. Rare individuals that produce flowers with anthers and stigmas located at the same height have been identified, and are self-compatible in most cases [9,10,11]. These “homostyle” individuals were originally considered to result from rare recombination events between alleles of the *S*-locus super-gene complex, and classical genetic analyses of these homostyles interpreted as indicating the presence of at least three genetic sub-components of the locus, termed *G* (female gynoecium characteristics), *P* (pollen size and incompatibility phenotype), and *A* (male androecium characteristics) [10,11].

Several major breakthroughs have recently revolutionized our understanding of the genetic control of HSI in *Primula* [12,13,14]. It is now clear that the *S*-locus is a hemizygous insertion found only in the thrum morph genome, and contains just five genes. As one of the *S*-locus genes (*PvGLO2*) has homology to MADS-box transcription factors, and another is likely involved in hormone regulation (*CYP734A50*), it is almost certain that many genes outside of the *S*-locus are under its control. Previous studies have reported transcriptomes of *Primula* species, but were focused on identifying morph or species-specific transcripts [13,15,16]. No previous report has assessed and/or compared differential expression at different developmental time points, hence we sought to perform a preliminary investigation to determine whether this might provide novel insight into the processes underpinning HSI in *Primula*. Examination of these downstream processes, controlled by the *S*-locus components, which ultimately lead to the phenotypes seen in HSI, is a necessary aspect for understanding the evolution of this breeding system in *Primula*.

Here, we utilize RNA-seq data from *P. vulgaris* pin and thrum flower buds at early and mature developmental stages, using floral bud size as a metric for overall flower development. These reads were mapped to our previously reported transcriptome [14] and used to examine differential expression (DE) of transcripts between the floral morphs at these developmental time points. This allowed us to begin to identify broad biological processes responsible for differential floral organ positioning, development, and self-incompatibly in *P. vulgaris*. Most significantly, a potential difference in the timing of pollen development between floral morphs was identified. 

## 2. Results

### 2.1. Floral Transcriptome Metrics and Homology Based Functional Annotation

The sequence data analyzed here were originally generated and assembled into a reference transcriptome in [14]. Briefly, it were derived from floral buds (sepals removed) at two developmental time points for each mating type. The first, 3–4 mm buds (corresponding to 11–14 days pre-anthesis), represents the point at which floral morphology starts to diverge between mating types [17,18]. The second time point, 10+ mm buds (1–3 days pre-anthesis), represents a stage late in development just prior to flower opening. These stages were chosen to provide insight into both the development of heteromorphic morphology, and the self-incompatibility system.

**Figure 1:**
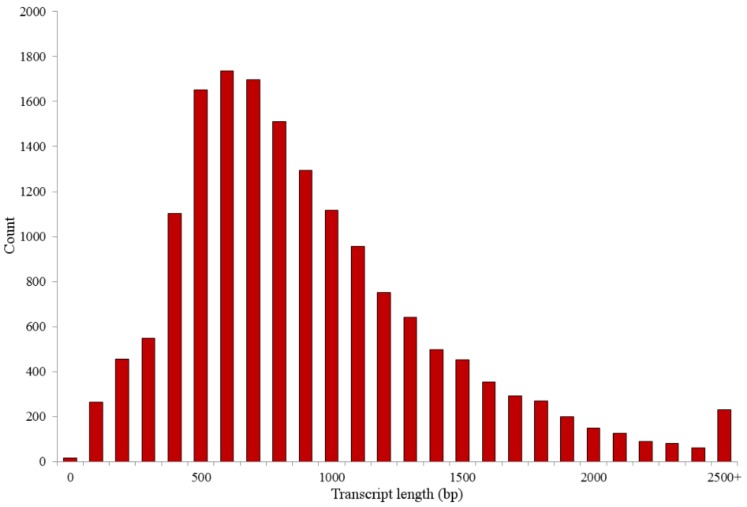
Distribution of transcript lengths. Bars represent counts of transcripts in each length category. Lengths are by hundreds, with 0 representing length of 0–99 bp and the scale progressing in this manner.

Previously unreported RNA-seq metrics with significance to this study are as follows: a total of 425.75 Mbp of sequence from 2,584,552 reads were generated. After quality filtering and adapter trimming, a total of 343.61 Mbp from 2,413,892 reads remained (Table 1). These reads were combined for de novo transcriptome assembly, yielding 16,544 unique transcript contigs with an average length of 963 bp, as previously described in [14]. Furthermore, previously unreported assembly metrics include a maximum transcript length of 8343 bp and a minimum length of 37 bp. The total length of all transcripts combined was approximately 15.9 Mbp and an N50 length of 1110 bp. The distribution of assembled transcript lengths is shown in Figure 1.

BLAST2GO software [19] was used to assign putative functions to transcripts based on sequence homology. Of the 16,544 transcripts, 14,821 (89.5%) had BLASTx hits with e-values less than 1.0 × 10^−10^ in the National Center for Biotechnology Information (NCBI) Non-Redundant Protein (nr) database. The remaining transcripts (~10%) are most likely either unique to *Primula vulgaris*, or too small to generate a significant match using BLASTx. Out of the transcripts with significant BLASTx scores, 13,767 (92.9%) were assigned putative Gene Ontology terms.

### 2.2. S-Locus Regulated Differential Expression

As a step towards identifying the large-scale processes and pathways involved in the development of heteromorphy and self-incompatibility, we calculated transcript abundance using the previously derived sequencing data. This was then used to assess differential expression of transcripts between pin and thrum morphs at the 3–4 mm and 10+ mm stages. Transcript expression levels were calculated by mapping RNA-seq read libraries from each morph and time point to the assembled transcriptome, and expression levels were normalized as RPKM (Reads Per Kilobase per Million). We considered transcripts with greater than or equal to three-fold difference in normalized expression and a Benjamini–Hochberg false discovery rate (FDR)-corrected *p*-value < 0.001 to be significantly differentially expressed. As the thrum morph genome should be the same as the pin, except for the hemizygous insertion of the *S*-locus, we considered any difference in expression between floral morphs to be resultant of the action of the *S*-locus. Therefore, transcripts more highly expressed in thrum flowers compared to pin flowers were considered *S*-locus up-regulated, while those with lower expression in thrum were considered *S*-locus down-regulated. Using these criteria, we identified a total of 540 transcripts as DE early in development and 3101 transcripts as DE at the later stage (Table 2). To assess the biological processes that these DE transcripts are involved in, we searched for Biological Process Gene Ontology (GO) terms that were over-represented (relative to the transcriptome as a whole) in the up- and down-regulated transcript sets. As high-level GO terms can be quite broad, we primarily examined Biological Process ontology terms in levels 4–6.

### 2.3. S-Locus Regulated Differential Expression in Young Flower Buds

In 3–4 mm thrum flower buds, 229 transcripts were identified as *S*-locus up-regulated, and 311 transcripts as *S*-locus down-regulated. A full table of these DE transcripts can be found in Supplementary Tables S1 and S2. At this stage, we detected expression of the *S*-locus gene *PvGLO2* in the up-regulated set, but we did not find evidence of expression for the other *S*-locus genes. 

**Figure 2:**
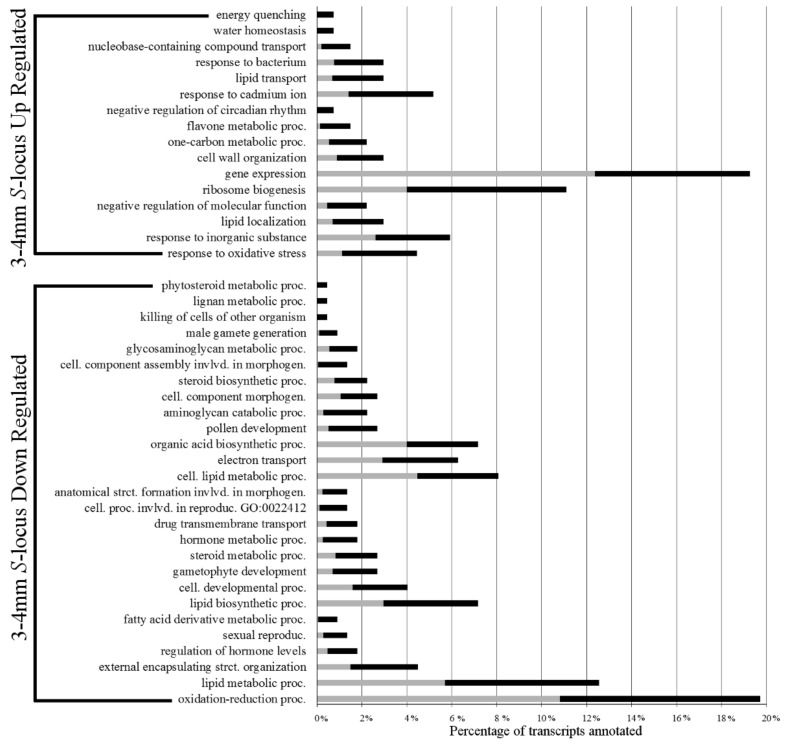
3–4 mm *S*-locus up- and down-regulated transcript over-represented biological process gene ontology terms. Grey bars represent percentage of all transcripts in the transcriptomes annotated with the respective term. Black bars represent the percentage of differential expression (DE) transcripts annotated with the respective term. The tips of the black bars represent the actual percentage observed.

Figure 2 shows over-represented Biological Process GO term annotations in levels 4–6 for these DE transcripts. The largest portion of *S*-locus up-regulated transcripts (nearly 20% in 3–4 mm buds) were annotated with gene expression related GO terms. At deeper ontology levels, we found more specific terms, such as negative regulation of gene expression and post-transcriptional gene silencing.

Early *S*-locus down-regulated transcripts were found to have a very different composition of over-represented GO terms. The largest class (~20%) was annotated with GO terms involved in oxidation-reduction processes. Likely related to these, a large number of transcripts involved in lipid and hormone metabolic processes were also present in this group. Upon closer examination at deeper ontology levels, several terms related to brassinosteroid metabolic processes were identified. One of the DE transcripts annotated with these terms was a homolog of the *Arabidopsis* gene *ROTUNDIFOLIA3*, a cytochrome P450 involved in brassinosteroid biosynthesis [20,21]. There was also over-representation of morphogenesis and sexual reproduction terms related to male functions in the set of *S*-locus down-regulated transcripts, such as gametophyte development, pollen development, pollen wall assembly, pollen exine formation, pollen sperm cell differentiation, and male gamete generation. A full list of over-represented GO terms can be found in Supplementary Tables S5 and S6

### 2.4. S-Locus Regulated Differential Expression in Mature Flower Buds

At the 10+ mm stage, 1489 transcripts were found to be up-regulated by the *S*-locus and 1612 down-regulated (see Supplementary Tables S3 and S4 for a full list of these DE transcripts). Again, one of the *S*-locus up-regulated transcripts was *PvGLO2*, but no other *S*-locus gene expression was detected. Interestingly, two of the most highly differentially expressed transcripts were homologs of small cysteine rich RALF (Rapid ALkalinization Factor) proteins. One of these had been previously identified as differentially expressed in *Primula* by suppressive subtractive hybridization [22]. These transcripts were expressed almost exclusively in the thrum morph. 

**Figure 3:**
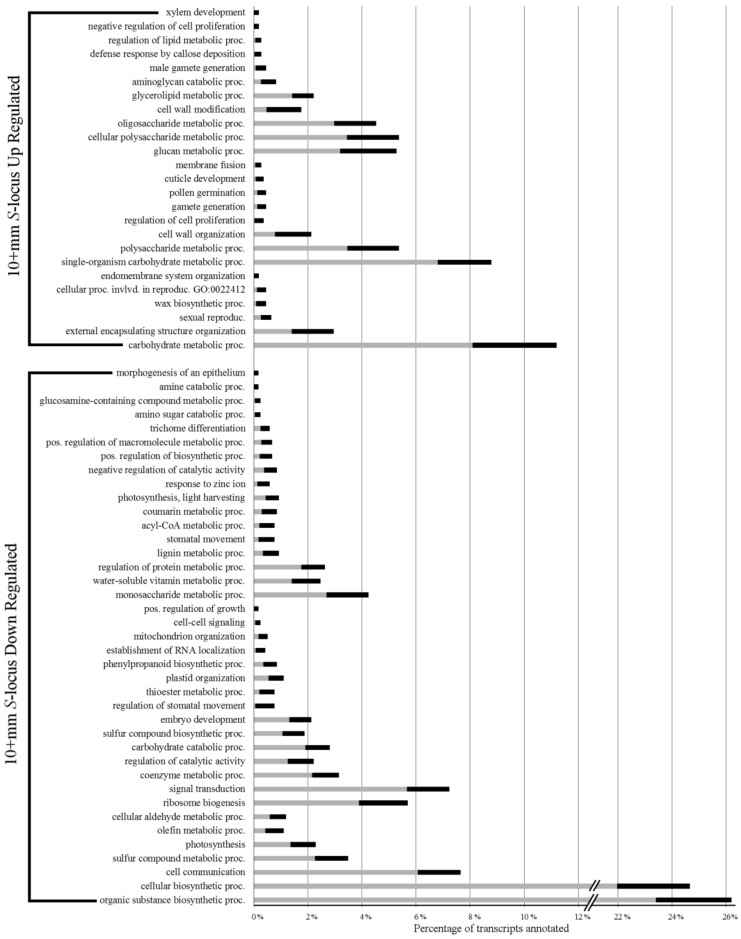
*S*-locus up- and down-regulated transcript over-represented biological process gene ontology terms. Grey bars represent percentage of all transcripts in the transcriptomes annotated with the respective term. Black bars represent the percentage of DE transcripts annotated with the respective term. The tips of the black bars represent the actual percentage observed.

Over-represented Biological Process GO terms in levels 4–6 are shown in Figure 3. Examination of *S*-locus up-regulated transcripts in 10+ mm buds revealed a sizable portion (~11%) annotated with carbohydrate metabolic process related terms. Likely associated with these were several over-represented terms related to cell growth, such as external encapsulating structure organization, wax biosynthesis process, cell wall organization, and cell wall modification. In contrast to the early developmental stage, sexual reproduction terms, such as sexual reproduction, cellular process involved in reproduction, gamete generation, pollen germination, and male gamete generation, were all over-represented in this up-regulated set. Finally, unlike the 3–4 mm *S*-locus up-regulated transcript set, over-representation of gene expression related terms was not seen in the genes up-regulated by the *S*-locus at this later stage. 

In the 10+ mm *S*-locus down-regulated set, we found the largest percentage of transcripts was annotated with terms related to biosynthetic processes. Unfortunately, it is not readily clear which specific process might be covered by this rather broad term. Cellular communication terms, including cell communication, signal transduction, and cell–cell signaling, were also found to be over-represented. The only developmental process that was over-represented in the down-regulated transcript set was embryo development. Upon examination of deeper ontology terms, we found several related to gene regulation, including both gene silencing and positive regulation of gene expression (see Supplementary Tables S7 and S8 for the full list of over-represented GO terms).

## 3. Discussion

### 3.1. S-Locus Regulated Differential Expression Changes throughout Floral Development

When comparing the two stages of development, far more differentially expressed transcripts were found later in development (3101) than early in development (540). To interpret this result, we considered several factors. First, the 3–4 mm early flower stage is the first point at which developmental differences become noticeable between the two morphs [17,18]. Second, the *S*-locus is known to be a hemizygous insertion found only in the thrum morph genome consisting of only five genes [12,13,14]. With so few genes contained within the *S*-locus, a cascade of gene expression changes is the most likely cause of the large change in DE observed between the two stages. This interpretation is supported by the over-representation of gene regulation terms in the early *S*-locus up-regulated gene set (Figure 2). Furthermore, there were a large number of transcripts with expression significantly lower in the thrum morph than in the pin. This included transcripts such as alpha dioxygenases (C5009 and C7996) and a cytochrome P450 (C6628), which were found at fairly high levels exclusively in the pin morph. As the presence/absence of the *S*-locus region is the difference between the morphs and is absent from the pin genotype, this suggests that it plays a role—either directly or indirectly—in negative regulation of gene expression and silencing. 

Previously, a *RALF-like* transcript was identified as DE between pin and thrum flowers [22]. Here, we found two different *RALF-like* transcripts almost exclusively expressed in the thrum morph. Critically, these *RALF-like* genes are not found in the *S*-locus, indicating that they are instead up-regulated by the effects of the *S*-locus at the later developmental stage. RALF proteins are a family of small, secreted, cysteine rich peptides implicated in numerous developmental processes, particularly cell elongation and expansion (reviewed in [23]). In floral tissues, they have primarily been found in pollen, and in *Solanum lycopersicum* and *Arabidopsis thaliana*, they inhibit pollen tube elongation and germination, respectively [24,25,26]. In *Arabidopsis*, a RALF has been identified as a ligand for the receptor-like kinase FERONIA, and their interaction has been shown to inhibit cell elongation in roots [27]. Tantalizingly, FERONIA also plays a critical role in plant reproduction, specifically signaling between pollen tubes and synergid cells [28]. These observations make these *RALF-like* transcripts interesting candidates for involvement in various aspects of both floral morphology and/or a self-incompatibility reaction. 

### 3.2. Expression of S-Locus Genes and Their Possible Roles in S-Locus Controlled Differential Expression

Out of the five *S*-locus genes previously identified [12,13,14], *PvGLO2* was the only one with detectable expression within our dataset. While a detailed time course expression analysis of *S*-locus genes has not yet been carried out, there are two possible explanations for the lack of detection. One possibility is that they are not expressed at these time points. The second is that they are expressed at very low levels below the sensitivity of our methods. The latter possibility is supported by previously reported expression data, in which *PvGLO2* was expressed at a dramatically higher level than the other four *S*-locus genes, albeit at an unreported time point [13]. The data in the current work suggest that expression of *PvGLO2* spans a broad period of time, from early to late floral development. As PvGLO2 shows homology to MADS-box transcription factors and CYP734A50 is likely involved in brassinosteroid regulation, these genes represent strong candidates for setting up the large-scale shift in gene expression observed between the early and late time points. 

### 3.3. Brassinosteroid Related Differences in Expression

The proposed mechanism of action for the *S*-locus gene CYP734A50 is through degradation of brassinosteroids (BR), causing a reduction in cell expansion in developing pistil cells, and leading to the short style found in the thrum morph [12]. In view of this, it was interesting that we found an over-representation of brassinosteroid and hormone related GO terms in the early down-regulated gene set. One of these was a homolog of *Arabidopsis ROTUNDIFOLIA3*, a cytochrome P450 thought to convert typhasterol to castasterone [20,21]. Castasterone levels have been found to be significantly lower in styles from thrum flowers than from pin flowers [12]. Our finding that this gene is down-regulated in thrum buds suggests several possibilities, the first being that the degradation of BR by CYP734A50 could cause a feedback down-regulation of genes involved in BR synthesis and regulation. The second is that another gene in the *S*-locus may have co-evolved to down-regulate expression of these genes, reinforcing the short style phenotype. Alternatively, BR may be acting in other organs besides the style. There is a precedent for such a role in relation to breeding systems. It has recently been reported that higher than optimal BR limits petal-cell proliferation in self-compatible *Capsella rubella*, leading to a smaller petal size than in the outcrossing species *C. grandiflora* [29]. These possibilities will need to be investigated in greater detail on an organ-specific level.

### 3.4. Pollen Development Timing Differences

Intriguingly, we found that pollen development related genes were differentially expressed at both early and late stages. Early in floral development, we found they were down-regulated by the *S*-locus, while later in development, they were up-regulated. This is suggestive of pollen developmental program differences between the pin and thrum morphs when compared with the other floral organs, with pollen development being delayed in thrum. This finding could underlie some of the differences observed between pin and thrum pollen, with pin producing roughly twice as many pollen grains, but those grains being only half the size (volume) of those of thrum pollen [30,31]. Furthermore, with the *S*-locus components and structure now known, it appears likely that the mechanisms of self-incompatibility differ between the morphs. Indeed, this has previously been suggested because of the very different sites of SI action that are observed between the two morphs [2,3,32,33,34]. Taken together, these data lead us to hypothesize that self-incompatibility may be a result of broader physiological differences (incongruities) between pollen types and stigmas/styles, rather than the allelic “lock and key” type recognition system seen in homomorphic SI systems [35]. For several reasons, we speculate that the *S*-locus gene initiating the observed differences in pollen related gene expression is *PvGLO2*. First, *PvGLO2* has homology to *GLOBOSA* type B-functional MADS-box transcription factors, and hence is likely to directly impact transcription of other genes. Second, our preliminary investigations have found *PvGLO2* to be primarily expressed in the anthers and petals (Burrows and McCubbin, manuscript in preparation). Finally, transposon insertion into *PvGLO2* has been correlated with the development of short homostyly [13]. This last finding has implicated *PvGLO2* in the control of anther height. While we find this highly likely in this analysis, as the transcriptomes were from whole buds, we are unable to identify specific downstream pathways though which *PvGLO2* might control anther height. Future RNA-seq, using specific tissues or comparing thrum and short homostyle flower buds or stamen, will be necessary to address this.

## 4. Materials and Methods 

### 4.1. Plant Material, RNA Extraction, RNA-Seq, and Transcriptome Assembly

For more details, see [14]. Briefly, a population of *P. vulgaris* plants were grown under greenhouse conditions. Floral buds (excluding sepals) were collected from both mating types at two developmental time points (3–4 mm and 10+ mm bud sizes), flash frozen, and stored at −80 °C. For each sample, 4 g of floral buds from six independent plants were used for RNA extraction and subjected to mRNA purification prior to library construction. Sequencing libraries for each mRNA sample were constructed from 200 ng of polyA mRNA using the Rapid RNA Library Kit (Roche, Basel, Germany), and sequenced with a Roche 454 FLX titanium (Roche, Basel, Germany). To increase sequencing depth in the young bud samples, mRNA from 3–4 mm thrum and 3–4 mm pin samples was also used to generate Ion Torrent sequencing libraries using the Ion Total RNA Seq V.2 kit (Life Technologies, Carlsbad, CA, USA), and sequenced with an Ion Torrent PGM. All library construction and sequencing was performed by the Washington State University Genomics Core Laboratory. Sequencing reads were trimmed to remove adapters, quality filtered using default parameters, and then used for de novo transcriptome assembly. Assembly was performed using the SeqMan NGen assembler (DNAStar, Madison, WI, USA), with default parameters (match size 21, match spacing 75, minimum match percentage 85%).

### 4.2. Transcriptome Annotation

Annotation of the reference transcriptome was carried out using the BLAST2GO software package [19]. Using BLAST2GO, a BLASTx search of the NCBI non-redundant protein (nr) database was run for each transcript using a minimum e-value of 1.0 × 10^−10^ and a “max hits” of 15. Gene Ontology term annotation of the transcripts was then performed using default settings.

### 4.3. Transcript Abundance and Differential Expression Analysis

Transcript abundance calculations and differential expression analysis were performed using CLC Genomics Workbench (CLCBio). Read mapping was performed using default parameters and transcript abundance was normalized as Reads Per Kilobase per Million. Two DE analyses were performed; one comparing expression between 3–4 mm pin and thrum flower buds, and a second comparing expression of 10+ mm pin and thrum flower buds. Sequences with three-fold or greater difference in expression and a Benjamini–Hochberg false discovery rate (FDR)-corrected *p*-value < 0.001 were considered significantly differentially expressed. All differential expression, the differences between thrum and pin, was considered to be a result of the actions of the *S*-locus. Putative functions of DE transcripts were considered based on GO terms annotated to them. For each up- and down-regulated set of transcripts, BLAST2GO was used to identify Biological Function GO terms that were over-represented when compared with the transcriptome as a whole. A *p*-value < 0.05 filter was used to identify over-represented GO terms. 

### 4.4. Data Availability 

Sequence read data is available at the NCBI, BioProject Accession number PRJNA294594. Specific gene sequences discussed in this manuscript have been deposited in GenBank.

## 5. Conclusions

Our findings here, coupled with the new findings about the nature and composition of the *Primula S*-locus [12,13,14], suggest at the very least, that the actions of the *S*-locus lead to large-scale transcriptional differences between the morphs throughout floral development. Furthermore, analysis of differentially expressed transcripts forms a basis for proposing hypotheses regarding the mechanisms behind HSI. These include the potential for additional roles for brassinosteroid signaling beyond controlling style length, and that differential expression of pollen development genes may contribute with the observed differences in pollen number, morphology, and potentially self-incompatibility. The results of this preliminary study suggest that detailed examination of *Primula* pollen development in both morphs is warranted, as well as transcriptomic investigation of homostyle flowers to examine how each *S*-locus component contributes to the observed transcriptomic differences.

## Figures and Tables

**Table 1:** Summary of sequencing statistics for *P. vulgaris* pin and thrum flower buds.

Sequencing Platform	454 FLX Titanium				Ion Torrent PGM	
Sample	Thrum 4 mm	Pin 4 mm	Thrum 10 mm	Pin 10 mm	Thrum 4 mm	Pin 4 mm
Total Read Number	69,930	87,277	296,829	279,923	923,071	927,522
Total Bases (bp)	21,674,904	27,179,118	97,703,248	91,824,090	92,086,555	95,282,501
Avg. Read Length (bp)	310	311	329	328	100	103
Read Number After Trimming	69,910	87,228	296,714	279,706	854,549	825,785
Total Bases After Trimming (bp)	21,221,593	26,575,047	95,986,850	90,107,495	56,402,359	53,316,852
Avg. Length After Trimming (bp)	304	305	323	322	66	65

**Table 2:** Differentially expressed transcript summary.

Category	Transcript Number
3–4 mm *S*-locus Down ^1^	311
3–4 mm *S*-locus Up ^1^	229
10+ mm *S*-locusDown ^2^	1612
10+ mm *S*-locus Up ^2^	1489

^1^ Total number of transcripts detected at this stage was 16,255; ^2^ Total number of transcripts detected at this stage was 15,095.

## Supplementary Materials

The following are available online at http://www.mdpi.com/2223-7747/7/2/38/s1, Table S1: 3–4 mm *S*-locus up-regulated transcripts, Table S2: 3–4 mm *S*-locus down-regulated transcripts, Table S3: 10+ mm *S*-locus up-regulated transcripts, Table S4: 10+ mm *S*-locus down-regulated transcripts, Table S5: 3–4 mm *S*-locus up-regulated GO terms, Table S6: 3–4 mm *S*-locus down-regulated GO terms, Table S7: 10+ mm *S*-locus up-regulated GO terms, Table S8: 10+ mm *S*-locus down-regulated GO terms.
